# Inhibition of proteasome activity by the dietary flavonoid apigenin is associated with growth inhibition in cultured breast cancer cells and xenografts

**DOI:** 10.1186/bcr1797

**Published:** 2007-11-21

**Authors:** Di Chen, Kristin R Landis-Piwowar, Marina S Chen, Q Ping Dou

**Affiliations:** 1 The Prevention Program, Barbara Ann Karmanos Cancer Institute and Department of Pathology, School of Medicine, Wayne State University, Detroit, Michigan 48201-2013, USA

## Abstract

**Introduction:**

Proteasome inhibition is an attractive approach to anticancer therapy and may have relevancy in breast cancer treatment. Natural products, such as dietary flavonoids, have been suggested as natural proteasome inhibitors with potential use for cancer prevention and therapeutics. We previously reported that apigenin, a flavonoid widely distributed in many fruits and vegetables, can inhibit proteasome activity and can induce apoptosis in cultured leukemia Jurkat T cells. Whether apigenin has proteasome-inhibitory activity in the highly metastatic human breast MDA-MB-231 cells and xenografts, however, is unknown.

**Methods:**

MDA-MB-231 breast cancer cell cultures and xenografts were treated with apigenin, followed by measurement of reduced cellular viability/proliferation, proteasome inhibition, and apoptosis induction. Inhibition of the proteasome was determined by levels of the proteasomal chymotrypsin-like activity, by ubiquitinated proteins, and by accumulation of proteasome target proteins in extracts of the treated cells or tumors. Apoptotic cell death was measured by capase-3/caspase-7 activation, poly(ADP-ribose) polymerase cleavage, and immunohistochemistry for terminal nucleotidyl transferase-mediated nick end labeling positivity.

**Results:**

We report for the first time that apigenin inhibits the proteasomal chymotrypsin-like activity and induces apoptosis not only in cultured MDA-MB-231 cells but also in MDA-MB-231 xenografts. Furthermore, while apigenin has antibreast tumor activity, no apparent toxicity to the tested animals was observed.

**Conclusion:**

We have shown that apigenin is an effective proteasome inhibitor in cultured breast cancer cells and in breast cancer xenografts. Furthermore, apigenin induces apoptotic cell death in human breast cancer cells and exhibits anticancer activities in tumors. The results suggest its potential benefits in breast cancer prevention and treatment.

## Introduction

Regular consumption of a variety of polyphenolic compounds has been associated with reduced cancer risk and with tumor growth suppression [1]. The polyphenolic flavone apigenin is widely distributed among fruits and vegetables, and apigenin has been shown to possess chemopreventive activities in a number of cancer models including those of lung cancer [2], skin cancer [3], cervical cancer [4], prostate cancer [5], and leukemia [6]. The mechanisms by which apigenin imparts its anticancer effects are varied and may include action through antiinflammation [7], free radical scavenging [8], and proteasome inhibition [6,9].

The eukaryotic proteasome is a large multicatalytic, multisubunit protease complex possessing at least three distinct activities, which are associated with three different β subunits, respectively: chymotrypsin-like activity (with the β_5 _subunit), trypsin-like activity (with the β_2 _subunit), and peptidyl-glutamyl peptide-hydrolyzing-like (caspase-like) activity (with the β_1 _subunit) [10]. Inhibition of the chymotrypsin-like activity, but not of the trypsin-like activity, of the proteasome has been associated with induction of tumor cell apoptosis [11,12]. By examining a broad range of cell culture models, it has been found that proteasome inhibitors rapidly induce tumor cell apoptosis, selectively activate the cell death program in cancer or oncogene-transformed cells, but not in normal or untransformed cells, and are able to trigger apoptotic death in human cancer cells that are resistant to various anticancer agents [9,11,13-18].

The most described and best known proteasome inhibitor, PS-341 (bortezomib, Velcade^®^; Millenium Pharmaceuticals Inc., Cambridge, MA, USA and Johnson Pharmaceutical Research and Development, LLC, Raritan, NJ, USA), is a dipeptide boronic acid analog with the cell-death-inducing activity found in several tumor cell lines and animal models [19-21]. The mechanism of action of PS-341 has been shown to be inhibition of the β_5_-subunit and the β_1_-subunit, with the β5-subunit as the predominant cell-death inducing target [22].

Because clinically available proteasome inhibitors are associated with some toxicity [13,14], natural proteasome inhibitors with less or no toxicity are attractive potential anticancer agents. The pursuit for nontoxic natural compounds has been stimulated by our findings that apigenin potently inhibits the chymotrypsin-like activity of a purified 20S proteasome and 26S proteasome in cultured tumor leukemia cells [6,9]. Proteasome inhibition led to the accumulation of proteasome target proteins (such as IκBα and Bax) and to subsequent induction of apoptosis in human leukemia cancer cells, as measured by activation of caspases and cleavage of poly(ADP-ribose) polymerase (PARP) [6,9].

The chemopreventive effects of apigenin are well defined [23], and we have observed cytotoxic effects in leukemia cells [6,9]. Whether apigenin has potential antibreast cancer activity and whether it could target the breast cancer proteasome, however, remain unclear. In the current study, we provide evidence that the proteasome-inhibitory activity of apigenin extends to breast cancer cells and tumors. Proteasome inhibition, growth suppression, and apoptosis induction were observed in cultured breast cancer MDA-MB-231 cells treated with apigenin. Since our previous studies revealed that apigenin was innocuous to normal cells [6,9], treatment of breast-cancer-bearing nude mice with apigenin was examined – resulting in tumor growth inhibition and massive apoptosis induction, associated with proteasome inhibition *in vivo*. No apparent toxicity to the tested animals was observed. The data suggest that apigenin acts as a natural proteasome inhibitor under physiological conditions. While cancer prevention has been the predominant attribute assigned to apigenin, our findings are indicative of great potential for cancer treatment.

## Materials and methods

### Materials

Apigenin, bisbenzimide Hoechst number 33258 stain, 3-[4,5-dimethyltiazol-2-yl]-2.5-diphenyl-tetrazolium bromide (MTT), dimethylsulfoxide (DMSO), cremophor and other chemicals were purchased from Sigma-Aldrich (St Louis, MO, USA). RPMI 1640, penicillin, and streptomycin were purchased from Invitrogen (Carlsbad, CA, USA). The fluorogenic peptide substrates Suc-LLVY-AMC (for the proteasomal chymotrypsin-like activity) and *N*-acetyl-DEVD-AMC (for caspase-3/caspase-7 activity) were from Calbiochem (San Diego, CA, USA). Mouse monoclonal antibody against human PARP was purchased from BIOMOL International LP (Plymouth Meeting, PA, USA). Mouse monoclonal antibodies against Bax (B-9), ubiquitin (P4D1), goat polyclonal antibody against actin (C-11), rabbit polyclonal antibody against IκBα (C15), and secondary antibodies were from Santa Cruz Biotechnology, Inc. (Santa Cruz, CA, USA). The antibody of p27 for immunohistochemistry was from Novocastra Laboratories Ltd (Newcastle upon Tyne, UK).

### Cell culture and cell extract preparation

MDA-MB-231 cells were grown in RPMI 1640 supplemented with 10% fetal bovine serum, 100 u/ml penicillin, and 100 μg/ml streptomycin. Cells were grown at 37°C in a humidified incubator with an atmosphere of 5% CO_2_. A whole-cell extract was prepared as previously described [24]. Briefly, MDA-MB-231 breast cancer cells were grown to 60–70% confluency, and were treated with 25 μM, 50 μM, 75 μM, or 100 μM apigenin or DMSO control (at a concentration equivalent to the volume used for the highest concentration of apigenin or ≤ 0.1%) for 24 hours. At the endpoint of the experiments, cells were harvested, washed twice with PBS and homogenized in a lysis buffer (50 mM Tris (pH 8.0), 5 mM ethylenediamine tetraacetic acid, 150 mM NaCl, 0.5% NP40). After 30 minutes of rocking at 4°C, the mixtures were centrifuged at 12,000 × *g *for 15 minutes and the supernatants were collected as whole-cell extracts.

### Proteasomal chymotrypsin-like and caspase-3/caspase-7 activity assays

Whole-cell extracts (10 μg) of cells treated with apigenin or tumor tissue extracts (10 μg) from human breast tumor xenograft were incubated for 2.5 hours at 37°C in 100 μl assay buffer (50 mmol/l Tris–HCl, pH 7.5) with 10 μmol/l fluorogenic substrate Suc-LLVY-AMC (for proteasomal chymotrypsin-like activity) or Ac-DEVD-AMC (for caspase-3/caspase-7 activity) as described previously [25]. After incubation, production of free hydrolyzed 7-amino-4-methylcoumarin (AMC) groups liberated by substrate hydrolysis was fluorometrically measured using a Victor 3 Multilabel Counter with an excitation filter of 380 nm and an emission filter of 460 nm (PerkinElmer, Boston, MA, USA).

### Cell viability/proliferation assay

The MTT assay, an index of cell viability and cell growth, was used to determine the effects of various compounds on MDA-MB-231 breast cancer cells. Cells were plated in a 96-well plate and were grown to 70–80% confluence, followed by addition of each compound at the indicated concentrations. After 24 hours of incubation at 37°C, the cell viability/proliferation was measured as previously described [26]. All samples were assayed in triplicate in three independent experiments, and the mean value for each experiment was calculated. The results are displayed as the mean ± standard deviation and are expressed as percentage of the control, which was considered to be 100%.

### Cellular morphology analysis

A Zeiss (Thornwood, NY, USA) Axiovert 25 microscope was used for all microscopic imaging with either phase contrast for cellular morphology, as previously described [27].

### Western blot analysis

The whole-cell extracts or tumor lysates were separated by SDS-PAGE gel and were transferred to a nitrocellulose membrane. Western blot analysis was performed using specific antibodies against ubiquitin, Bax, IκBα, PARP or β-actin, followed by visualization using the enhanced chemiluminescence reagent, as previously described [27].

### Human breast tumor xenograft experiments

Female athymic nude mice, age 5 weeks, were purchased from Taconic Research Animal Services (Hudson, NY, USA) and were housed in accordance with protocols approved by the Institutional Laboratory Animal Care and Use Committee of Wayne State University. Human breast cancer MDA-MB-231 cells (5 × 10^6^) suspended in 0.1 ml serum-free RPMI 1640 were inoculated subcutaneously in both flanks of each mouse (four mice per group).

When the tumors reached sizes of ~120 mm^3 ^on average, the mice were randomly grouped (*n *= 4) and were treated by daily subcutaneously injection with 25 or 50 mg/kg apigenin, or vehicle (10% DMSO, 40% Cremophor/ethanol (3:1) and 50% PBS). The tumor size was measured every other day using calipers, and the tumor volumes were calculated according to a standard formula: length × width^2^/2. Mice were sacrificed after 29 days of treatment. The tumors were collected and photographed and the tumor tissues were used for multiple assays.

### Terminal nucleotidyl transferase-mediated nick end labeling and immunohistochemistry using tumor tissue samples

The terminal nucleotidyl transferase-mediated nick end labeling (TUNEL) assay using an *in situ *apoptosis detection kit, and immunostaining of p27 were performed as described previously [27]. The proteasomal chymotrypsin-like activity assay and western blot analysis using animal tumor samples were performed as described above using cultured breast cancer cells.

### Statistical analysis

To evaluate the difference between a treatment and control, the Student *t *test was applied. The level of significance was set at *P *< 0.05.

## Results

**Figure 1:**
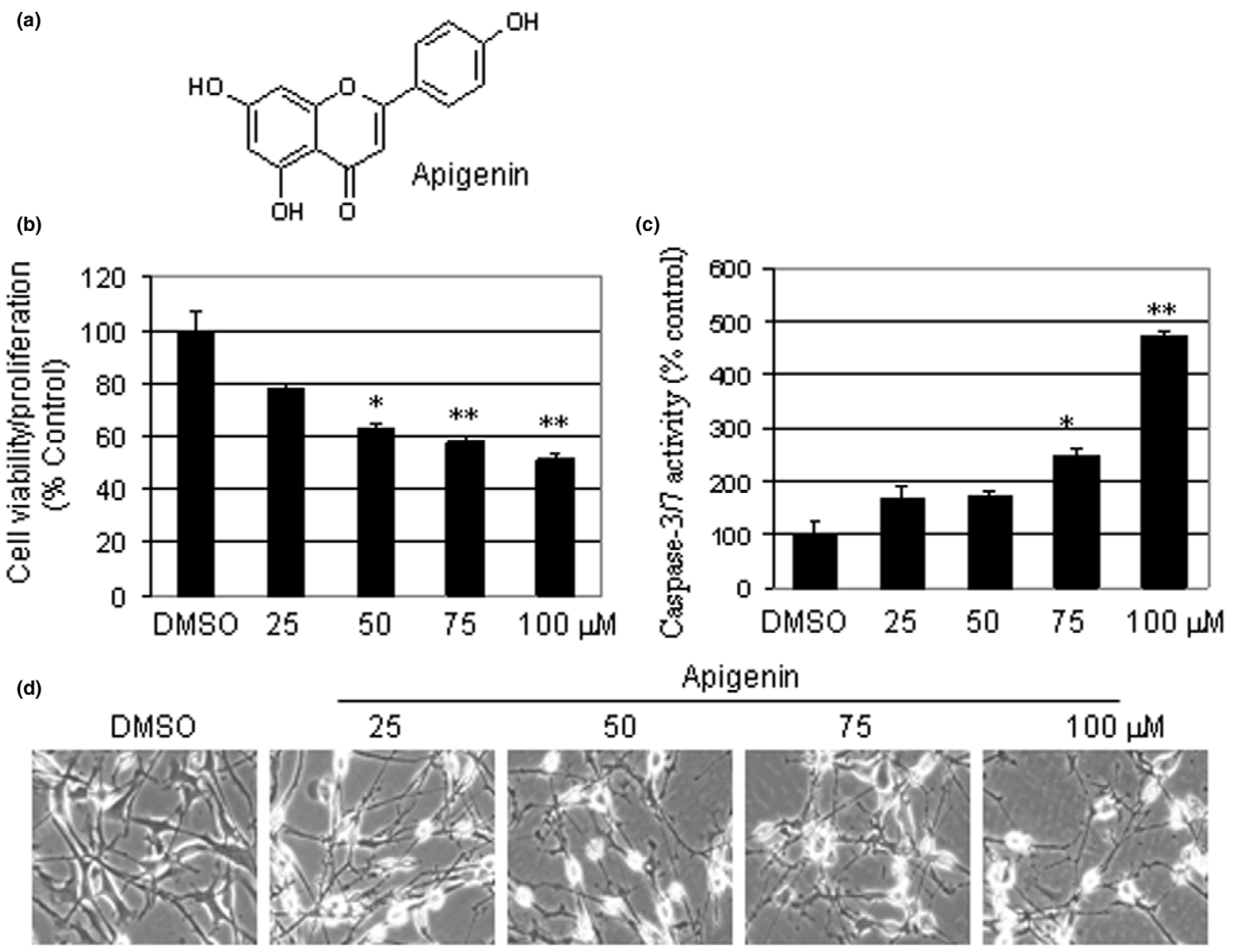
Antiproliferative and apoptosis inductive activities of apigenin. **(a) **Chemical structure of apigenin. **(b) **Viability/proliferation-inhibitory effect of apigenin on the MDA-MB-231 breast cancer cell line. MDA-MB-231 cells were treated for 24 hours with increasing concentrations of apigenin followed by a 3-[4,5-dimethylthiazol-2-yl]-2,5-diphenyl-tetrazolium bromide assay. **(c) **Activation of caspase-3/caspase-7 activity in MDA-MB-231 cells treated with indicated concentrations of apigenin for 24 hours, followed by measuring the caspase-3/caspase-7 activity in cell extracts. **(d) **Examination for morphological changes visualized by phase-contrast imaging (100× magnification). Three independent experiments were performed and each individual experiment was tested in triplicate ((b) and (c)). Similar results were found with all experiments and the data shown represent one experiment; bars, standard deviation. DMSO, dimethylsulfoxide. *P < 0.05, **P < 0.01.

Apigenin could inhibit cell viability/proliferation and could activate caspase-3/caspase-7 activity of MDA-MB-231 breast cancer cells in a dose-dependent manner. We previously showed that leukemia Jurkat T cells exposed to increasing concentrations of apigenin (up to 50 μM) were subject to up to 80% cell death after 24 hours of treatment [6]. In the present study, we first investigated the effects of apigenin (Figure 1a) in a dose-dependent manner (25, 50, 75, or 100 μM) on the highly metastatic and invasive human breast cancer cell line, MDA-MB-231 cells. The results of an MTT assay revealed that apigenin inhibited cell viability, and potentially proliferation, after 24 hours of treatment in a dose-dependent manner (Figure 1b). Compared with the DMSO control, treatment with 25 μM, 50 μM, 75 μM and 100 μM apigenin inhibited cell viability/proliferation by 12%, 27%, 42%, and 49%, respectively (Figure 1b). Furthermore, dose-dependent caspase-3/caspase-7 activation was observed in the same experiment with a nearly five-fold increase in the cells treated at the highest concentration (Figure 1c). Additionally, morphological changes were observed after increasing doses of apigenin in the same experiment (Figure 1d). Compared with the DMSO control, cells became elongated, losing their characteristic morphology and possibly indicating cellular stress (Figure 1d).

**Figure 2:**
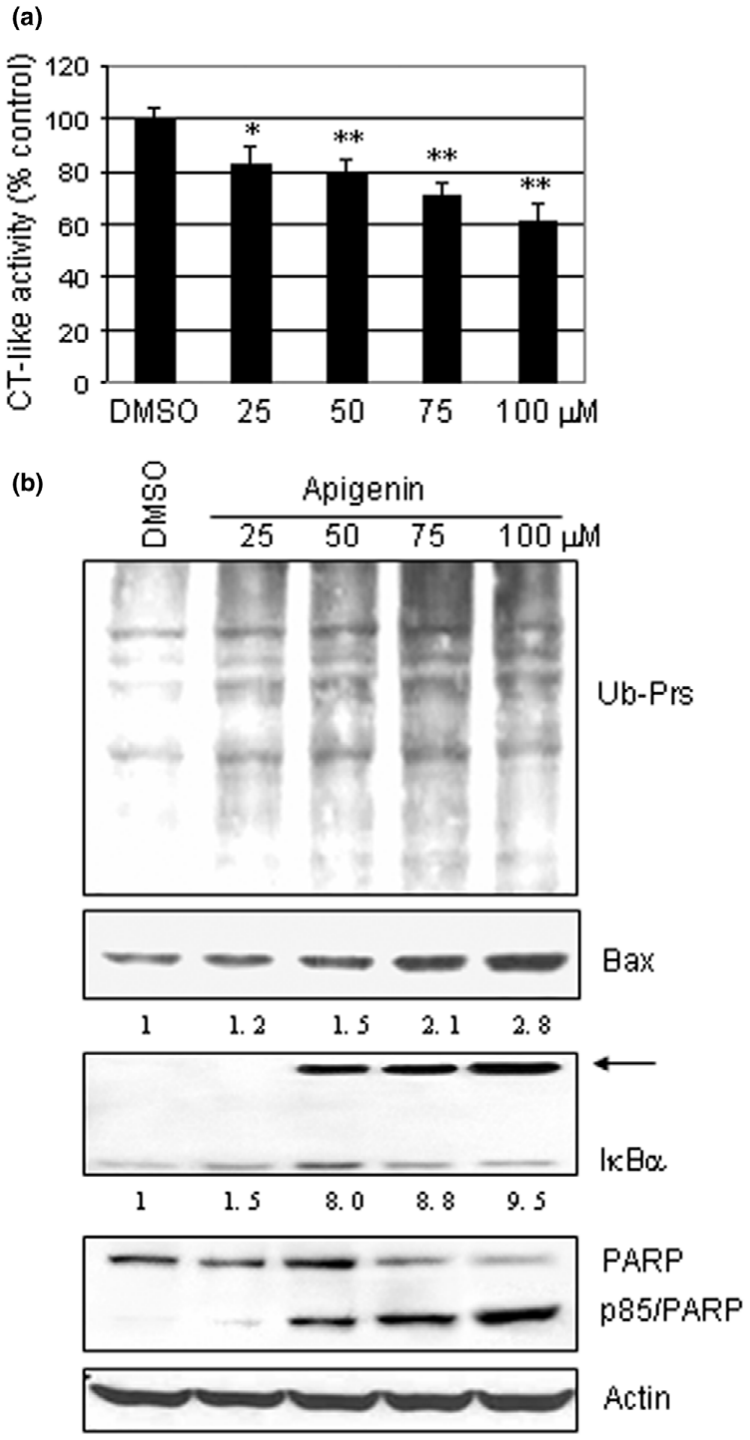
Apigenin-induced proteasomal inhibition and apoptosis in MDA-MB-231 breast cancer cells. **(a) **Inhibition of proteasomal chymotrypsin (CT)-like activity by apigenin in MDA-MB-231 cells. Cells were treated with various concentrations of apigenin for 24 hours, were harvested and were analyzed for the proteasomal chymotrypsin-like activity in cell extracts. **(b) **Western blot analysis for accumulation of ubiquitinated proteins (Ub-Prs), Bax, IκBα and poly(ADP-ribose) polymerase cleavage (PARP) in the above prepared cell extracts after 72 hours of treatment. Arrow, ubiquitinated IκBα (p56). Three independent experiments were performed and each individual experiment was tested in triplicate. Similar results were found with all experiments and the data shown represent one experiment; bars, standard deviation. DMSO, dimethylsulfoxide. *P < 0.05, **P < 0.01. The number under each band of the western blot indicates a quantitative analysis by densitometry. Quantitative number for IκBα included both bands of intact IκBα (p37) and ubiquitinated IκBα (p56).

We have previously shown that apigenin can inhibit the chymotrypsin-like proteasome activity in leukemia Jurkat T cells [6]. We therefore hypothesized that apigenin could likewise target the tumor cellular proteasome in MDA-MB-231 cells. To determine whether the reduced cell viability/proliferation by apigenin was due to its proteasome-inhibitory activity, MDA-MB-231 cells were treated with 25 μM, 50 μM, 75 μM, or 100 μM apigenin for 24 hours. After treatment, proteins were extracted and were used to measure the proteasome inhibition by the fluorogenic proteasomal chymotrypsin-like activity assay and by western blot analysis. The proteasomal chymotrypsin-like activity was inhibited by 17%, 20%, 29%, and 40% in the cells treated with 25 μM, 50 μM, 75 μM, and 100 μM apigenin, respectively (Figure 2a). Western blot analysis revealed an accumulation of ubiquitinated proteins (indicated by the increased intensity of the protein smear compared with the DMSO control) and the proteasome target protein Bax, most predominantly in cells exposed to the highest concentrations (Figure 2b). We have previously reported an ubiquitinated form of IκBα protein with a molecular weight of about 56 kDa [9]. A similar p56 band appeared after treatment of apigenin at 50–100 μM, detectable by the specific antibody to IκBα (Figure 2b, arrow).

Inhibition of the proteasomal chymotrypsin-like activity, but not trypsin-like activity, has been shown to be associated with apoptosis induction in cancer cells [11,12]. To investigate whether the proteasomal inhibition is associated with apoptosis induction, the presence of cleaved PARP was examined. Apoptosis-specific cleaved PARP was detected in cells treated with 50 μM apigenin and higher concentrations (Figure 2b). These results demonstrate that apigenin is able to inhibit the proteasomal chymotrypsin-like activity, resulting in apoptosis induction in human breast cancer MDA-MB-231 cells.

**Figure 3:**
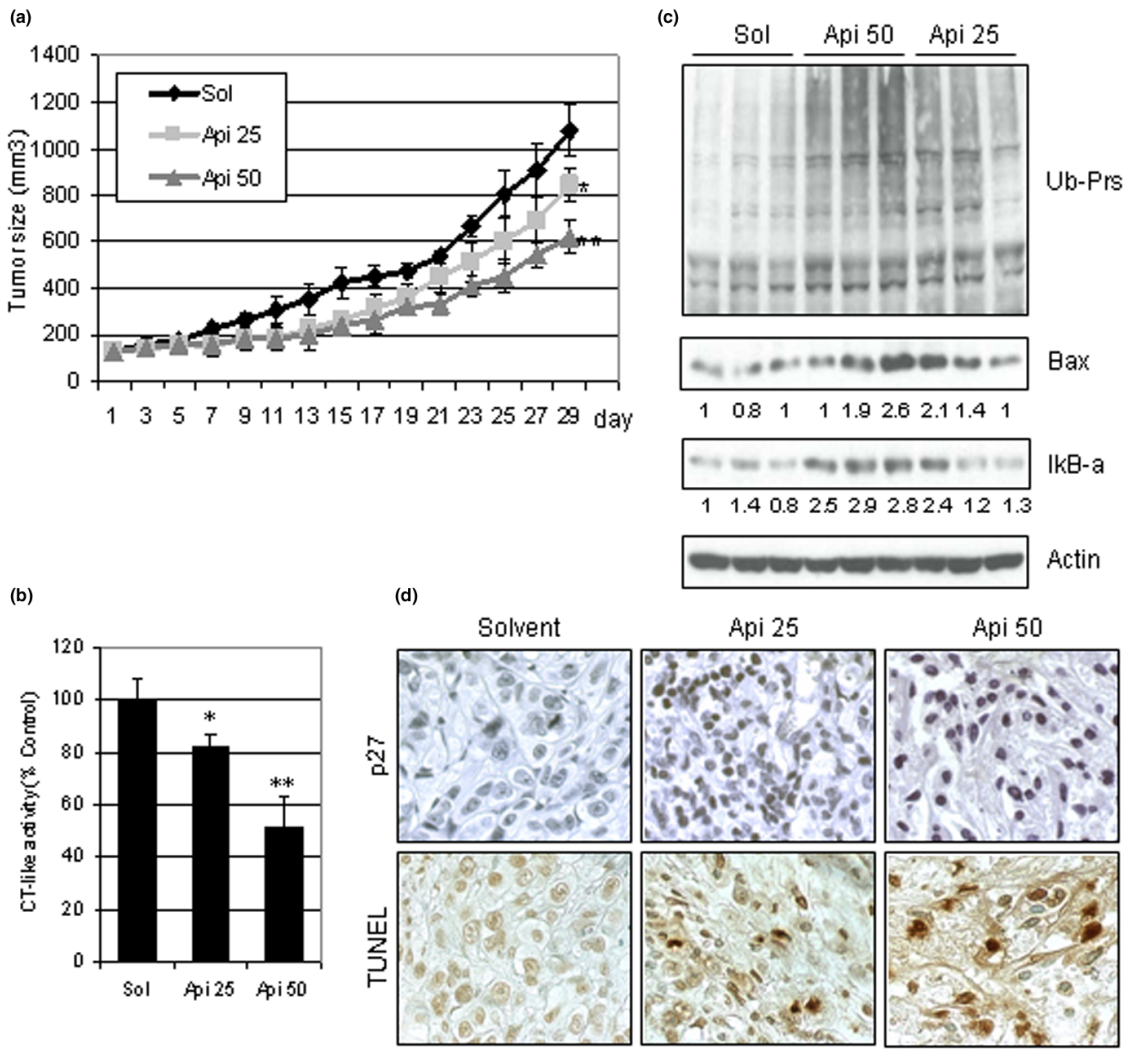
Apigenin antitumor activity associated with inhibition of proteasomal activity and induction of apoptosis *in vivo*. Female nude mice bearing MDA-MB-231 cell tumors were treated with the solvent control (Sol) or with 25 or 50 mg/kg/day apigenin (Api 25, Api 50) for 29 days. Tumors were collected after 29 days of treatment, and the prepared tissues were analyzed by multiple assays. **(a) **Inhibition of MDA-MB-231 tumor growth by apigenin. **(b) **Proteasomal chymotrypsin (CT)-like activity measured in the tumor samples. **(c) **Western blot analysis for accumulation of ubiquitinated proteins (Ub-Prs), Bax, IκBα and actin presented three representative individual tumor samples from each group of mice. **(d) **Immunohistochemistry for p27 and terminal nucleotidyl transferase-mediated nick end labeling (TUNEL). Data points, mean tumor volume in each experimental group containing four mice; bars, standard deviation. *P < 0.05, **P < 0.01. Number under each band of the western blot indicates a quantitative analysis by densitometry compared with the loading control of actin.

Apigenin inhibits the growth of human breast cancer xenografts, associated with proteasome inhibition and apoptosis induction *in vivo*. After we demonstrated that apigenin could inhibit proteasomal activity and induce apoptosis in cultured breast cancer cells (Figures 1 and 2), we then determined whether apigenin could exert antitumor activity and whether that activity is associated with proteasome inhibition and apoptosis induction. Breast cancer MDA-MB-231 cells (5 × 10^6^) were implanted subcutaneously in 5-week-old female athymic nude mice. Upon the tumors reaching a palpable size (~120 mm^3 ^on average), the mice were treated subcutaneously with either the vehicle control or with 25 or 50 mg/kg apigenin daily. Significant tumor growth inhibition by apigenin was observed after 29 days of treatment, demonstrating that apigenin has antitumor activity (Figure 3a). Control tumors grew to an average size of 1,077 ± 114 mm^3^, while 25 mg/kg apigenin-treated tumors grew to 842 ± 71 mm^3 ^and 50 mg/kg apigenin-treated tumors grew to 622 ± 66 mm^3^. This corresponds to 22% growth inhibition in the 25 mg/kg apigenin-treated tumors (*P *< 0.05; Figure 3a) and to 43% growth inhibition in the 50 mg/kg apigenin-treated tumors (*P *< 0.01; Figure 3a). We monitored the body weight of mice from each group, and the average body weight on the last day of treatment was 26.8 g, 26.5 g and 26.3 g from the mice treated with the solvent, with 25 mg/kg apigenin, or with 50 mg/kg apigenin, respectively. The data of the body weight suggested that there was no toxicity of apigenin observed in the treated mice.

At the end of the experiment, the MDA-MB-231 tumors were collected and used for proteasome activity assays and for western blot analysis. The proteasomal chymotrypsin-like activity was inhibited by ~20% and ~50% in the tumors treated with 25 mg/kg and 50 mg/kg apigenin, respectively, compared with the control mice (Figure 3b). Consistently in western blot analysis, the accumulation of IκBα protein and a trend of increased Bax protein expression were observed in tumors treated with apigenin, especially in the tumors treated with 50 mg/kg apigenin (Figure 3c). In addition, the immunohistochemistry results confirmed the increased expression of p27 only in the tumors treated with apigenin (Figure 3d). Apigenin therefore appears able to inhibit tumor proteasome activity and act as an antiproliferative agent *in vivo*.

To determine whether apoptosis is responsible for the observed antitumor activity of apigenin, immunohistochemistry of TUNEL was examined. The results revealed that an average of 10 ± 1% and 17 ± 2% of apoptotic cells, indicated by TUNEL positivity in four tumor slides of each group, were observed in tumors from animals treated with 25 mg/kg and 50 mg/kg apigenin, respectively (Figure 3d). There were some faint TUNEL-positive cells detected in the solvent-treated tumors (Figure 3d), indicating that this background was derived either from a solvent effect or from spontaneous tumor cell apoptosis. Taken together, these data show that the ability of apigenin to inhibit the proteasomal chymotrypsin-like activity is probably responsible for the observed induction of apoptosis in breast tumors *in vivo*.

## Discussion

Chemotherapy and radiotherapy have an important role as single modalities for cancer treatment; however, they largely have limited effectiveness in the treatment of solid human tumors that often become drug resistant and are often accompanied by severe drug-related side effects [28]. In an attempt to improve traditional therapies, the proteasome has become an increasingly important molecular target.

The antitumor and chemosensitizing properties of the proteasome inhibitor PS-341 are well documented [29]. Toxicity from bortezomib, however, has sparked an interest for identifying nontoxic natural proteasome inhibitors such as the flavonoid apigenin that could potentially serve as nontoxic therapeutic and chemopreventive strategies. A role for apigenin-induced proteasome inhibition has been substantiated in our previous work; apigenin and other flavonoids were shown to have proteasome-inhibitory activity in human tumor cells, but had little to no effect on normal, nontransformed cells [6,9].

We examined the effectiveness of apigenin in a breast cancer model using human MDA-MB-231 cells. After 24 hours, apigenin treatment inhibited breast cancer cell viability/proliferation by 50% with the highest concentration tested (Figure 1b). We have previously shown that apigenin is able to inhibit the chymotrypsin-like activity of a purified 20S proteasome [6]. We therefore hypothesized that the reduced cellular viability or possibly reduced proliferation was due in large part to apigenin's proteasomal inhibitory activity. To test this hypothesis, MDA-MB-231 cells were treated for 24 hours with apigenin, were harvested and lysed, and the cell extracts were examined for their chymotrypsin-like activity and by western blot analysis. At the highest concentration, proteasome activity was inhibited by approximately 40% (Figure 2a) and accumulated levels of ubiquitinated proteins, Bax and ubiquitinated IκBα were observed (Figure 2b). It is likely that an extended treatment in this *in vitro *model would provide even more striking data.

To ensure that the apparent proteasome inhibition by apigenin treatment would lead to the induction of apoptosis, we examined the morphological changes, the event of activated caspase-3/caspase-7 activity and, ultimately, the production of the cleaved PARP fragment. MDA-MB-231 cells exposed to apigenin exhibited changes in cellular morphology that may indicate a cellular stress (Figure 1d). A true measure of apoptosis in apigenin-treated cells was observed by caspase-3/caspase-7 activation and cleaved PARP most noticeably after treatment with the highest concentration of apigenin (Figures 1c and 2b). Again, extended treatments may be warranted for these apoptotic studies as well.

Because the *in vitro *findings in this epithelial cancer model were so promising, we then examined the effects of apigenin treatment in a mouse model. Apigenin has been shown to have growth-inhibitory effects associated with cell-cycle regulation in nude mice bearing prostate cancer tumors [30,31]. In our current study, treatment with apigenin resulted in significant inhibition of proteasomal chymotrypsin-like activity, accumulation of proteasome target proteins (that is, Bax), and induction of apoptosis in tumors (Figure 3). Associated with the observed proteasome inhibition and apoptosis induction, significant tumor growth inhibition (~43%) by apigenin at the highest concentration was observed in this breast tumor model (Figure 3a).

In the present work we have shown that apigenin, at relatively high doses, exhibits no gross toxicity in an animal model while exerting proteasome-inhibitory and growth-inhibitory effects both *in vitro *and *in vivo*. This finding is of particular importance for future studies, which may include the delivery of apigenin in combination with other natural compounds or as a sensitizing agent to traditional cancer therapeutics. In fact, a recent study suggested that apigenin may sensitize leukemia cells, prostate cancer cells, and colon cancer cells by inducing the expression of death receptor 5, which, in combination with exogenous tumor necrosis factor-related apoptosis-inducing ligand, acts synergistically to induce apoptosis while having no effect on normal cells [32]. The evidence from these studies suggests that apigenin has great potential to be further developed as a cancer therapeutic.

The concentrations of apigenin used to treat cultured breast cancer cells and used in the animal model are most probably higher than those found in the blood of individuals consuming dietary apigenin. The dose for apigenin in this mouse model, however, appeared to be well tolerated. During the 29-day treatment, there was no overall gross toxicity observed. More detailed microscopic and macroscopic pathologic studies are required to definitively document the lack of toxicity when apigenin is used at these concentrations.

## Conclusion

The importance of the proteasome in the pathological state of cancer and the potential for apigenin and other flavonoids as natural inhibitors of the proteasome may provide a viable modality for the prevention and treatment of some cancers. The findings reported in the present article indicate that apigenin appears to be a promising, novel anticancer agent, and one of its mechanisms of action involves targeting the tumor cellular proteasome and inducing apoptotic cell death. Using an innocuous natural product or its prodrug as a chemosensitizer may reduce the toxicity and boost the effectiveness of current chemotherapeutics. Future analysis of apigenin in a combinational regimen may elucidate increased effectiveness of this novel agent in additional cancer models.

## Abbreviations

AMC = 7-amino-4-methylcoumarin; DMSO = dimethylsulfoxide; MTT = 3-[4,5-dimethylthiazol-2-yl]-2,5-diphenyl-tetrazolium bromide; PARP = poly(ADP-ribose) polymerase; PBS = phosphate-buffered saline; TUNEL = terminal nucleotidyl transferase-mediated nick end labeling.

## Competing interests

The authors declare that they have no competing interests.

## Authors' contributions

DC and KRL-P contributed equally to this manuscript. DC participated and performed in the design of the animal study, data collection and interpretation, and manuscript preparation. KRL-P participated in the study design for the *in vitro *work, data collection and interpretation, and manuscript preparation. MSC participated in the study design for the *in vitro *work and participated in data collection. QPD was responsible for the design of the study, data interpretation, and manuscript preparation as well as supervision of the project. All authors read and approved the final manuscript.
